# Logistic regression model training based on the approximate homomorphic encryption

**DOI:** 10.1186/s12920-018-0401-7

**Published:** 2018-10-11

**Authors:** Andrey Kim, Yongsoo Song, Miran Kim, Keewoo Lee, Jung Hee Cheon

**Affiliations:** 1 Department of Mathematical Sciences, Seoul National University, 1 Gwanak-ro, Gwanak-gu, Seoul, 08826 Republic of Korea; 2 Department of Computer Science and Engineering, University of California- San Diego, 9500 Gillman Drive, San Diego, 92093-0404 California USA; 3 Division of Biomedical Informatics, University of California- San Diego, 9500 Gillman Drive, San Diego, 92093-0728 California USA

**Keywords:** Homomorphic encryption, Machine learning, Logistic regression

## Abstract

**Background:**

Security concerns have been raised since big data became a prominent tool in data analysis. For instance, many machine learning algorithms aim to generate prediction models using training data which contain sensitive information about individuals. Cryptography community is considering secure computation as a solution for privacy protection. In particular, practical requirements have triggered research on the efficiency of cryptographic primitives.

**Methods:**

This paper presents a method to train a logistic regression model without information leakage. We apply the homomorphic encryption scheme of Cheon et al. (ASIACRYPT 2017) for an efficient arithmetic over real numbers, and devise a new encoding method to reduce storage of encrypted database. In addition, we adapt Nesterov’s accelerated gradient method to reduce the number of iterations as well as the computational cost while maintaining the quality of an output classifier.

**Results:**

Our method shows a state-of-the-art performance of homomorphic encryption system in a real-world application. The submission based on this work was selected as the best solution of Track 3 at iDASH privacy and security competition 2017. For example, it took about six minutes to obtain a logistic regression model given the dataset consisting of 1579 samples, each of which has 18 features with a binary outcome variable.

**Conclusions:**

We present a practical solution for outsourcing analysis tools such as logistic regression analysis while preserving the data confidentiality.

**Electronic supplementary material:**

The online version of this article (10.1186/s12920-018-0401-7) contains supplementary material, which is available to authorized users.

## Background

Machine learning (ML) is a class of methods in artificial intelligence, the characteristic feature of which is that they do not give the solution of a particular problem but they learn the process of finding solutions to a set of similar problems. The theory of ML appeared in the early 60’s on the basis of the achievements of cybernetics [1] and gave the impetus to the development of theory and practice of technically complex learning systems [2]. The goal of ML is to partially or fully automate the solution of complicated tasks in various fields of human activity.

The scope of ML applications is constantly expanding; however, with the rise of ML, the security problem has become an important issue. For example, many medical decisions rely on logistic regression model, and biomedical data usually contain confidential information about individuals [3] which should be treated carefully. Therefore, privacy and security of data are the major concerns, especially when deploying the outsource analysis tools.

There have been several researches on secure computation based on cryptographic primitives. Nikolaenko et al. [4] presented a privacy preserving linear regression protocol on horizontally partitioned data using Yao’s garbled circuits [5]. Multi-party computation technique was also applied to privacy-preserving logistic regression [6–8]. However, this approach is vulnerable when a party behaves dishonestly, and the assumption for secret sharing is quite different from that of outsourcing computation.

Homomorphic encryption (HE) is a cryptosystem that allows us to perform certain arithmetic operations on encrypted data and receive an encrypted result that corresponds to the result of operations performed in plaintext. Several papers already discussed ML with HE techniques. Wu et al. [9] used Paillier cryptosystem [10] and approximated the logistic function using polynomials, but it required an exponentially growing computational cost in the degree of the approximation polynomial. Aono et al. [11] and Xie et al. [12] used an additive HE scheme to aggregate some intermediate statistics. However, the scenario of Aono et al. relies on the client to decrypt these intermediary statistics and the method of Xie et al. requires expensive computational cost to calculate the intermediate information. The most related research of this paper is the work of Kim et al. [13] which also used HE based ML. However, the size of encrypted data and learning time were highly dependent on the number of features, so the performance for a large dataset was not practical in terms of storage and computational cost.

Since 2011, the iDASH Privacy and Security Workshop has assembled specialists in privacy technology to discuss issues that apply to biomedical data sharing, as well as main stakeholders who provided an overview of the main uses of the data, different laws and regulations, and their own views on privacy. In addition, it has began to hold annual competitions on the basis of the workshop from 2014. The goal of this challenge is to evaluate the performance of state-of-the-arts methods that ensures rigorous data confidentiality during data analysis in a cloud environment.

In this paper, we provide a solution to the third track of iDASH 2017 competition, which aims to develop HE based secure solutions for building a ML model (i.e., logistic regression) on encrypted data. We propose a general practical solution for HE based ML that demonstrates good performance and low storage costs. In practice, our output quality is comparable to the one of an unencrypted learning case. As a basis, we use the HE scheme for approximate arithmetic [14]. To improve the performance, we apply several additional techniques including a packing method, which reduce the required storage space and optimize the computational time. We also adapt Nesterov’s accelerated gradient [15] to increase the speed of convergence. As a result, we could obtain a high-accuracy classifier using only a small number of iterations.

We give an open-source implementation [16] to demonstrate the performance of our HE based ML method. With our packing method we can encrypt the dataset with 1579 samples and 18 features using 39MB of memory. The encrypted learning time is about six minutes. We also demonstrate our implementation on the datasets used in [13] to compare the results. For example, the training of a logistic regression model took about 3.6 min with the storage about 0.02GB compared to 114 min and 0.69GB of Kim et al. [13] when a dataset consists of 1253 samples, each of which has 9 features.

## Methods

### Logistic regression

Logistic regression or logit model is a ML model used to predict the probability of occurrence of an event by fitting data to a logistic curve [17]. It is widely used in various fields including machine learning, biomedicine [18], genetics [19], and social sciences [20].

Throughout this paper, we treat the case of a binary dependent variable, represented by ± 1. Learning data consists of pairs (**x**_*i*_,*y*_*i*_) of a vector of co-variates ${\mathbf {x}}_{i} = (x_{i1},..., x_{if}) \in {\mathbb {R}}^{f}$ and a dependent variable *y*_*i*_∈{±1}. Logistic regression aims to find an optimal ${\boldsymbol {\beta }} \in {\mathbb {R}}^{f+1}$ which maximizes the likelihood estimator

$$\prod_{i=1}^{n} \Pr(y_{i}|{\mathbf{x}}_{i}) = \prod_{i=1}^{n}\frac{1}{1 + \exp(-y_{i}(1,{\mathbf{x}}_{i})^{T} {\boldsymbol{\beta}})},$$

or equivalently minimizes the loss function, defined as the negative log-likelihood:

$$J({\boldsymbol{\beta}}) = \frac{1}{n}\sum_{i=1}^{n} \log\left(1 + \exp\left(-\mathbf{z}_{i}^{T} {\boldsymbol{\beta}}\right)\right)$$

where **z**_*i*_=*y*_*i*_·(1,**x**_*i*_) for *i*=1,…,*n*.

### Gradient descent

Gradient Descent (GD) is a method for finding a local extremum (minimum or maximum) of a function by moving along gradients. To minimize the function in the direction of the gradient, one-dimensional optimization methods are used.

For logistic regression, the gradient of the cost function with respect to ***β*** is computed by

$$\nabla J({\boldsymbol{\beta}}) = -\frac{1}{n}\sum_{i=1}^{n} \sigma\left(-\mathbf{z}_{i}^{T} {\boldsymbol{\beta}}\right) \cdot \mathbf{z}_{i}$$

where $\sigma (x) = \frac {1}{1+\exp (-x)}$. Starting from an initial ***β***_0_, the gradient descent method at each step *t* updates the regression parameters using the equation

$${\boldsymbol{\beta}}^{(t+1)} \leftarrow {\boldsymbol{\beta}}^{(t)} + \frac{\alpha_{t}}{n}\sum_{i=1}^{n} \sigma\left(-\mathbf{z}_{i}^{T} {\boldsymbol{\beta}}^{(t)}\right) \cdot \mathbf{z}_{i} $$

where *α*_*t*_ is a learning rate at step *t*.

### Nesterov’s accelerated gradient

The method of GD can face a problem of zig-zagging along a local optima and this behavior of the method becomes typical if it increases the number of variables of an objective function. Many GD optimization algorithms are widely used to overcome this phenomenon. Momentum method, for example, dampens oscillation using the accumulated exponential moving average for the gradient of the loss function.

Nesterov’s accelerated gradient [15] is a slightly different variant of the momentum update. It uses moving average on the update vector and evaluates the gradient at this “looked-ahead” position. It guarantees a better rate of convergence *O*(1/*t*^2^) (vs. *O*(1/*t*) of standard GD algorithm) after *t* steps theoretically, and consistently works slightly better in practice. Starting with a random initial **v**_0_=***β***_0_, the updated equations for Nesterov’s Accelerated GD are as follows:

1 $$\begin{array}{*{20}l} \left\{\begin{array}{ll} {\boldsymbol{\beta}}^{(t+1)} & = {\mathbf{v}}^{(t)} - \alpha_{t} \cdot \bigtriangledown J\left({\mathbf{v}}^{(t)}\right), \\ {\mathbf{v}}^{(t+1)} & = (1-\gamma_{t})\cdot{\boldsymbol{\beta}}^{(t + 1)} + \gamma_{t}\cdot{\boldsymbol{\beta}}^{(t)}, \end{array}\right. \end{array} $$

where 0<*γ*_*t*_<1 is a moving average smoothing parameter.

### Approximate homomorphic encryption

HE is a cryptographic scheme that allows us to carry out operations on encrypted data without decryption. Cheon et al. [14] presented a method to construct a HE scheme for arithmetic of approximate numbers (called HEAAN in what follows). The main idea is to treat an encryption noise as part of error occurring during approximate computations. That is, an encryption *ct* of message $m \in {\mathcal {R}}$ by a secret key *sk* for a ciphertext modulus *q* will have a decryption structure of the form 〈*ct*,*sk*〉=*m*+*e* (mod *q*) for some small *e*.

The following is a simple description of HEAAN based on the ring learning with errors problem. For a power-of-two integer *N*, the cyclotomic polynomial ring of dimension *N* is denoted by ${\mathcal R} ={\mathbb {Z}} /\left (X^{N} + 1\right)$. For a positive integer *ℓ*, we denote ${\mathcal {R}}_{\ell } = {\mathcal {R}} / 2^{\ell } {\mathcal {R}} = {\mathbb {Z}}_{2^{\ell }}[\!X] /\left (X^{N} + 1\right)$ the residue ring of ${\mathcal {R}}$ modulo 2^*ℓ*^.

- KeyGen(1^*λ*^).
  - For an integer *L* that corresponds to the largest ciphertext modulus level, given the security parameter *λ*, output the ring dimension *N* which is a power of two.
  - Set the small distributions *χ*_key_,*χ*_err_,*χ*_enc_ over ${\mathcal R}$ for secret, error, and encryption, respectively.
  - Sample a secret *s*←*χ*_key_, a random $a\leftarrow {\mathcal R}_{L}$ and an error *e*←*χ*_err_. Set the secret key as *sk*←(1,*s*) and the public key as $\mathsf {pk}\leftarrow (b,a)\in {\mathcal R}_{L}^{2}$ where *b*←−*as*+*e* (mod 2^*L*^).
- KSGen_*sk*_(*s*^′^). For $s'\in {\mathcal R}$, sample a random $a^{\prime }\leftarrow {\mathcal R}_{2 \cdot L}$ and an error *e*^′^←*χ*_err_. Output the switching key as $\mathsf {swk}\leftarrow (b^{\prime },a^{\prime })\in {\mathcal R}_{2\cdot L}^{2}$ where *b*^′^←−*a*^′^*s*+*e*^′^+2^*L*^*s*^′^(mod 2^2·*L*^).
  - Set the evaluation key as *evk*←KSGen_*sk*_(*s*^2^).
- Enc_*pk*_(*m*). For $m\in {\mathcal R}$, sample *v*←*χ*_enc_ and *e*_0_,*e*_1_←*χ*_err_. Output *v*·*pk*+(*m*+*e*_0_,*e*_1_) (mod 2^*L*^).
- Dec_*sk*_(*ct*). For $\mathsf {ct}= (c_{0},c_{1})\in {\mathcal R}_{\ell }^{2}$, output *c*_0_+*c*_1_·*s* (mod 2^*ℓ*^).
- Add(*ct*_1_,*ct*_2_). For $\mathsf {ct}_{1},\mathsf {ct}_{2}\in {\mathcal R}_{\ell }^{2}$, output *ct*_*add*_←*ct*_1_+*ct*_2_ (mod 2^*ℓ*^).
- CMult_*evk*_(*ct*;*c*). For $\mathsf {ct}\in {\mathcal R}_{\ell }^{2}$ and $a\in {\mathcal R}$, output *ct*^′^←*c*·*ct* (mod 2^*ℓ*^).
- Mult_*evk*_(*ct*_1_,*ct*_2_). For $\mathsf {ct}_{1}=(b_{1},a_{1}),\mathsf {ct}_{2}=(b_{2},a_{2})\in {\mathcal R}_{\ell }^{2}$, let (*d*_0_,*d*_1_,*d*_2_)=(*b*_1_*b*_2_,*a*_1_*b*_2_+*a*_2_*b*_1_,*a*_1_*a*_2_) (mod 2^*ℓ*^). Output *ct*_*mult*_←(*d*_0_,*d*_1_)+⌊2^−*L*^·*d*_2_·*evk*⌉ (mod 2^*ℓ*^).
- ReScale(*ct*;*p*). For a ciphertext $\mathsf {ct}\in {\mathcal R}_{\ell }^{2}$ and an integer *p*, output *ct*^′^←⌊2^−*p*^·*ct*⌉ (mod 2^*ℓ*−*p*^).

For a power-of-two integer *k*≤*N*/2, HEAAN provides a technique to pack *k* complex numbers in a single polynomial using a variant of the complex canonical embedding map ${\phi :{\mathbb {C}}^{k} \rightarrow {\mathcal {R}}}$. We restrict the plaintext space as a vector of real numbers throughout this paper. Moreover, we multiply a scale factor of 2^*p*^ to plaintexts before the rounding operation to maintain their precision.

- Encode(**w**;*p*). For $\mathbf {w} \in {\mathbb {R}}^{k}$, output the polynomial $m \leftarrow \phi (2^{p}\cdot \mathbf {w})\in {\mathcal R}$.
- Decode(*m*;*p*). For a plaintext $m \in {\mathcal R}$, the encoding of an array consisting of a power of two *k*≤*N*/2 messages, output the vector $\mathbf {w} \leftarrow \phi ^{-1} (m / 2^{p}) \in {\mathbb {R}}^{k}$.

The encoding/decoding techniques support the parallel computation over encryption, yielding a better amortized timing. In addition, the HEAAN scheme provides the rotation operation on plaintext slots, i.e., it enables us to securely obtain an encryption of the shifted plaintext vector (*w*_*r*_,…,*w*_*k*−1_,*w*_0_,…,*w*_*r*−1_) from an encryption of (*w*_0_,…,*w*_*k*−1_). It is necessary to generate an additional public information *rk*, called the rotation key. We denote the rotation operation as follows.

- Rotate_*rk*_(*ct*;*r*). For the rotation keys *rk*, output a ciphertext *ct*^′^ encrypting the rotated plaintext vector of *ct* by *r* positions.

Refer [14] for the technical details and noise analysis.

### Database encoding

For an efficient computation, it is crucial to find a good encoding method for the given database. The HEAAN scheme supports the encryption of a plaintext vector and the slot-wise operations over encryption. However, our learning data is represented by a matrix (*z*_*ij*_)_1≤*i*≤*n*,0≤*j*≤*f*_. A recent work [13] used the column-wise approach, i.e., a vector of specific feature data (*z*_*ij*_)_1≤*i*≤*n*_ is encrypted in a single ciphertext. Consequently, this method required (*f*+1) number of ciphertexts to encrypt the whole dataset.

In this subsection, we suggest a more efficient encoding method to encrypt a *matrix* in a single ciphertext. A training dataset consists of *n* samples $\mathbf {z}_{i}\in {\mathbb {R}}^{f+1}$ for 1≤*i*≤*n*, which can be represented as a matrix *Z* as follows:

$$Z=\left[\begin{array}{cccc} z_{10} & z_{11} & \cdots & z_{1f} \\ z_{20} & z_{21} & \cdots & z_{1f} \\ \vdots & \vdots & \ddots & \vdots \\ z_{n0} & z_{n1} & \cdots & z_{nf} \end{array}\right].$$

For simplicity, we assume that *n* and (*f*+1) are power-of-two integers satisfying log*n*+ log(*f*+1)≤ log(*N*/2). Then we can pack the whole matrix in a single ciphertext in a row-by-row manner. Specifically, we will identify this matrix with the *k*-dimensional vector by (*z*_*ij*_)_1≤*i*≤*n*,0≤*j*≤*f*_↦**w**=(*w*_*ℓ*_)_0≤*ℓ*<*n*·(*f*+1)_ where *w*_*ℓ*_=*z*_*ij*_ such that *ℓ*=(*f*+1)(*i*−1)+*j*, that is,

$$Z\mapsto \mathbf{w}=(z_{10},\dots,z_{1f},z_{20},\dots,z_{2f},\dots,z_{n0},\dots,z_{nf}).$$

In a general case, we can pad zeros to set the number of samples and the dimension of a weight vector as powers of two.

It is necessary to perform shifting operations of row and column vectors for the evaluation of the GD algorithm. In the rest of this subsection, we explain how to perform these operations using the rotation algorithm provided in the HEAAN scheme. As described above, the algorithm Rotate(*ct*;*r*) can shift the encrypted vector by *r* positions. In particular, this operation is useful in our implementation when *r*=*f*+1 or *r*=1. For the first case, a given matrix *Z*=(*z*_*ij*_)_1≤*i*≤*n*,0≤*j*≤*f*_ is converted into the matrix

$$Z'=\left[\begin{array}{cccc} z_{20} & z_{21} & \cdots & z_{2f} \\ \vdots & \vdots & \ddots & \vdots \\ z_{n0} & z_{n1} & \cdots & z_{nf} \\ z_{10} & z_{11} & \cdots & z_{1f} \end{array}\right],$$

while the latter case outputs the matrix

$$Z^{\prime\prime}=\left[\begin{array}{cccc} z_{11} & \cdots & z_{1f} & z_{20} \\ z_{21} & \cdots & z_{2f} & z_{30} \\ \vdots & \vdots & \ddots & \vdots \\ z_{n1} & \cdots & z_{nf} & z_{10} \end{array}\right]$$

over encryption. The matrix *Z*^′^ is obtained from *Z* by shifting its row vectors and *Z*^′′^ can be viewed as an *incomplete* column shifting because of its last column.

### Polynomial approximation of the sigmoid function

One limitation of the existing HE cryptosystems is that they only support polynomial arithmetic operations. The evaluation of the sigmoid function is an obstacle for the implementation of the logistic regression since it cannot be expressed as a polynomial.

Kim et al. [13] used the least squares approach to find a global polynomial approximation of the sigmoid function. We adapt this approximation method and consider the degree 3, 5, and 7 least squares polynomials of the sigmoid function over the domain [−8,8]. We observed that the inner product values $\mathbf {z}_{i}^{T}{\boldsymbol {\beta }}^{(t)}$ in our experimentations belong to this interval. For simplicity, a least squares polynomial of *σ*(−*x*) will be denoted by *g*(*x*) so that we have $g\left (\mathbf {z}_{i}^{T}{\boldsymbol {\beta }}^{(t)}\right)\approx \sigma \left (-\mathbf {z}_{i}^{T}{\boldsymbol {\beta }}^{(t)}\right)$ when $\left |\mathbf {z}_{i}^{T}{\boldsymbol {\beta }}^{(t)}\right |\le 8$. The approximate polynomials *g*(*x*) of degree 3, 5, and 7 are computed as follows:

$$\left\{\begin{array}{ll} g_{3}(x) & = 0.5-1.20096\cdot(x/8)+0.81562\cdot(x/8)^{3},\\ g_{5}(x) & = 0.5-1.53048\cdot(x/8)+2.3533056\cdot(x/8)^{3} \\ & \ \ \ -1.3511295\cdot(x/8)^{5}, \\ g_{7}(x) & = 0.5-1.73496\cdot(x/8)+4.19407\cdot(x/8)^{3} \\ & \ \ \ -5.43402\cdot(x/8)^{5}+2.50739\cdot(x/8)^{7}.\\ \end{array}\right.$$

A low-degree polynomial requires a smaller evaluation depth while a high-degree polynomial has a better precision. The maximum errors between *σ*(−*x*) and the least squares *g*_3_(*x*), *g*_5_(*x*), and *g*_7_(*x*) are approximately 0.114, 0.061 and 0.032, respectively.

### Homomorphic evaluation of the gradient descent

This section explains how to securely train the logistic regression model using the HEAAN scheme. To be precise, we explicitly describe a full pipeline of the evaluation of the GD algorithm. We adapt the same assumptions as in the previous section so that the whole database can be encrypted in a single ciphertext.

First of all, a client encrypts the dataset and the initial (random) weight vector ***β***^(0)^ and sends them to the public cloud. The dataset is encoded to a matrix *Z* of size *n*×(*f*+1) and the weight vector is copied *n* times to fill the plaintext slots. The plaintext matrices of the resulting ciphertexts are described as follows:

$$\begin{aligned} \mathsf{ct}_{z}=& \texttt{Enc}\left[\begin{array}{cccc} z_{10} & z_{11} & \cdots & z_{1f} \\ z_{20} & z_{21} & \cdots & z_{1f} \\ \vdots & \vdots & \ddots & \vdots \\ z_{n0} & z_{n1} & \cdots & z_{nf} \end{array}\right],\\ \\ \mathsf{ct}_{\beta}^{(0)}=& \texttt{Enc}\left[\begin{array}{cccc} \beta_{0}^{(0)} & \beta_{1}^{(0)} & \cdots & \beta_{f}^{(0)} \\ \beta_{0}^{(0)} & \beta_{1}^{(0)} & \cdots & \beta_{f}^{(0)} \\ \vdots & \vdots & \ddots & \vdots \\ \beta_{0}^{(0)} & \beta_{1}^{(0)} & \cdots & \beta_{f}^{(0)} \end{array}\right]. \end{aligned}$$

As mentioned before, both *Z* and ***β***^(0)^ are scaled by a factor of 2^*p*^ before encryption to maintain the precision of plaintexts. We skip to mention the scaling factor in the rest of this section since every step will return a ciphertext with the scaling factor of 2^*p*^.

The public server takes two ciphertexts *ct*_*z*_ and $\mathsf {ct}_{\beta }^{(t)}$ and evaluates the GD algorithm to find an optimal modeling vector. The goal of each iteration is to update the modeling vector ***β***^(*t*)^ using the gradient of loss function:

$${\boldsymbol{\beta}}^{(t+1)} \leftarrow {\boldsymbol{\beta}}^{(t)} + \frac{\alpha_{t}}{n}\sum_{i=1}^{n} \sigma\left(-\mathbf{z}_{i}^{T} {\boldsymbol{\beta}}^{(t)}\right) \cdot \mathbf{z}_{i}$$

where *α*_*t*_ denotes the learning rate at the *t*-th iteration. Each iteration consists of the following eight steps.

**Step 1:** For given two ciphertexts *ct*_*z*_ and $\mathsf {ct}_{\beta }^{(t)}$, compute their multiplication and rescale it by *p* bits:

$$\mathsf{ct}_{1} \leftarrow \texttt{ReScale}\left(\texttt{Mult}\left(\mathsf{ct}^{(t)}_{\beta}, \mathsf{ct}_{z}\right); p\right).\\ $$

The output ciphertext contains the values $z_{ij} \cdot \beta ^{(t)}_{j}$ in its plaintext slots, i.e.,

$$\mathsf{ct}_{1}=\texttt{Enc}\left[\begin{array}{cccc} z_{10}\cdot\beta_{0}^{(t)} & z_{11}\cdot\beta_{1}^{(t)} & \cdots & z_{1f}\cdot\beta_{f}^{(t)} \\ z_{20}\cdot\beta_{0}^{(t)} & z_{21}\cdot\beta_{1}^{(t)} & \cdots & z_{1f}\cdot\beta_{f}^{(t)} \\ \vdots & \vdots & \ddots & \vdots \\ z_{n0}\cdot\beta_{0}^{(t)} & z_{n1}\cdot\beta_{1}^{(t)} & \cdots & z_{nf}\cdot\beta_{f}^{(t)} \end{array}\right].$$

**Step 2:** To obtain the inner product $\mathbf {z}_{i}^{T} {\boldsymbol {\beta }}^{(t)}$, the public cloud aggregates the values of $z_{ij}\beta _{j}^{(t)}$ in the same row. This step can be done by adapting the incomplete column shifting operation.

One simple way is to repeat this operation (*f*+1) times, but the computational cost can be reduced down to log(*f*+1) by adding *ct*_1_ to its rotations recursively:

$$\mathsf{ct}_{1} \leftarrow \texttt{Add}\left(\mathsf{ct}_{1}, \texttt{Rotate}\left(\mathsf{ct}_{1}; 2^{j}\right)\right),$$

for *j*=0,1,…, log(*f*+1)−1. Then the output ciphertext *ct*_2_ encrypts the inner product values $\mathbf {z}_{i}^{T}{\boldsymbol {\beta }}^{(t)}$ in the first column and some “garbage” values in the other columns, denoted by ⋆, i.e.,

$$\mathsf{ct}_{2}=\texttt{Enc}\left[\begin{array}{cccc} \mathbf{z}_{1}^{T}{\boldsymbol{\beta}}^{(t)} & \star & \cdots & \star \\ \mathbf{z}_{2}^{T}{\boldsymbol{\beta}}^{(t)} & \star & \cdots & \star \\ \vdots & \vdots & \ddots & \vdots \\ \mathbf{z}_{n}^{T}{\boldsymbol{\beta}}^{(t)} & \star & \cdots & \star \end{array}\right].$$

**Step 3:** This step performs a constant multiplication in order to annihilate the garbage values. It can be obtained by computing the encoding polynomial *c*←Encode(*C*;*p*_*c*_) of the matrix

$$C=\left[\begin{array}{cccc} 1 & 0 & \cdots & 0 \\ 1 & 0 & \cdots & 0 \\ \vdots & \vdots & \ddots & \vdots \\ 1 & 0 & \cdots & 0 \end{array}\right],$$

using the scaling factor of $\phantom {\dot {i}\!}2^{p_{c}}$ for some integer *p*_*c*_. The parameter *p*_*c*_ is chosen as the bit precision of plaintexts so it can be smaller than the parameter *p*.

Finally we multiply the polynomial *c* to the ciphertext *ct*_2_ and rescale it by *p*_*c*_ bits:

$$\begin{array}{*{20}l} \mathsf{ct}_{3} &\leftarrow \texttt{ReScale}(\texttt{CMult}(\mathsf{ct}_{2}; c); p_{c}). \end{array} $$

The garbage values are multiplied with zero while one can maintain the inner products in the plaintext slots. Hence the output ciphertext *ct*_3_ encrypts the inner product values in the first column and zeros in the others:

$$\mathsf{ct}_{3}=\texttt{Enc}\left[\begin{array}{cccc} \mathbf{z}_{1}^{T}{\boldsymbol{\beta}}^{(t)} & 0 & \cdots & 0 \\ \mathbf{z}_{2}^{T}{\boldsymbol{\beta}}^{(t)} & 0 & \cdots & 0 \\ \vdots & \vdots & \ddots & \vdots \\ \mathbf{z}_{n}^{T}{\boldsymbol{\beta}}^{(t)} & 0 & \cdots & 0 \end{array}\right].$$

**Step 4:** The goal of this step is to replicate the inner product values to other columns. Similar to Step 2, it can be done by adding the input ciphertext to its column shifting recursively, but in the opposite direction

$$\mathsf{ct}_{3} \leftarrow \texttt{Add}\left(\mathsf{ct}_{3}, \texttt{Rotate}\left(\mathsf{ct}_{3}; -2^{j}\right)\right)$$

for *j*=0,1,…, log(*f*+1)−1. The output ciphertext *ct*_4_ has the same inner product value in each row:

$$\mathsf{ct}_{4}=\texttt{Enc}\left[\begin{array}{cccc} \mathbf{z}_{1}^{T}{\boldsymbol{\beta}}^{(t)} & \mathbf{z}_{1}^{T}{\boldsymbol{\beta}}^{(t)} & \cdots & \mathbf{z}_{1}^{T}{\boldsymbol{\beta}}^{(t)} \\ \mathbf{z}_{2}^{T}{\boldsymbol{\beta}}^{(t)} & \mathbf{z}_{2}^{T}{\boldsymbol{\beta}}^{(t)} & \cdots & \mathbf{z}_{2}^{T}{\boldsymbol{\beta}}^{(t)} \\ \vdots & \vdots & \ddots & \vdots \\ \mathbf{z}_{n}^{T}{\boldsymbol{\beta}}^{(t)} & \mathbf{z}_{n}^{T}{\boldsymbol{\beta}}^{(t)} & \cdots & \mathbf{z}_{n}^{T}{\boldsymbol{\beta}}^{(t)} \end{array}\right].$$

**Step 5:** This step simply evaluates an approximating polynomial of the sigmoid function, i.e., *ct*_5_←*g*(*ct*_4_) for some *g*∈{*g*_3_,*g*_5_,*g*_7_}. The output ciphertext encrypts the values of $g\left (\mathbf {z}_{i}^{T}{\boldsymbol {\beta }}^{(t)}\right)$ in its plaintext slots:

$$\mathsf{ct}_{5}=\texttt{Enc}\left[\begin{array}{ccc} g\left(\mathbf{z}_{1}^{T}{\boldsymbol{\beta}}^{(t)}\right) & \cdots & g\left(\mathbf{z}_{1}^{T}{\boldsymbol{\beta}}^{(t)}\right) \\ g\left(\mathbf{z}_{2}^{T}{\boldsymbol{\beta}}^{(t)}\right) & \cdots & g\left(\mathbf{z}_{2}^{T}{\boldsymbol{\beta}}^{(t)}\right) \\ \vdots & \ddots & \vdots \\ g\left(\mathbf{z}_{n}^{T}{\boldsymbol{\beta}}^{(t)}\right) & \cdots & g\left(\mathbf{z}_{n}^{T}{\boldsymbol{\beta}}^{(t)}\right) \end{array}\right].$$

**Step 6:** The public cloud multiplies the ciphertext *ct*_5_ with the encrypted dataset *ct*_*z*_ and rescales the resulting ciphertext by *p* bits:

$$\begin{array}{*{20}l} \mathsf{ct}_{6} &\leftarrow \texttt{ReScale}(\texttt{Mult}(\mathsf{ct}_{5}, \mathsf{ct}_{z}); p). \end{array} $$

The output ciphertext encrypts the *n* vectors $g\left (\mathbf {z}_{i}^{T}{\boldsymbol {\beta }}^{(t)}\right)\cdot \mathbf {z}_{i}$ in each row:

$$\mathsf{ct}_{6}=\texttt{Enc}\left[\begin{array}{ccc} g\left(\mathbf{z}_{1}^{T}{\boldsymbol{\beta}}^{(t)}\right)\cdot z_{10} & \cdots & g\left(\mathbf{z}_{1}^{T}{\boldsymbol{\beta}}^{(t)}\right)\cdot z_{1f} \\ g\left(\mathbf{z}_{2}^{T}{\boldsymbol{\beta}}^{(t)}\right)\cdot z_{20} & \cdots & g\left(\mathbf{z}_{2}^{T}{\boldsymbol{\beta}}^{(t)}\right)\cdot z_{2f} \\ \vdots & \ddots & \vdots \\ g\left(\mathbf{z}_{n}^{T}{\boldsymbol{\beta}}^{(t)}\right) \cdot z_{n0} & \cdots & g\left(\mathbf{z}_{n}^{T}{\boldsymbol{\beta}}^{(t)}\right)\cdot z_{nf} \end{array}\right].$$

**Step 7:** This step aggregates the vectors $g\left (\mathbf {z}_{i}^{T}{\boldsymbol {\beta }}^{(t)}\right)$ to compute the gradient of the loss function. It is obtained by recursively adding *ct*_6_ to its row shifting:

$$\mathsf{ct}_{6} \leftarrow \texttt{Add}\left(\mathsf{ct}_{6}, \texttt{Rotate}\left(\mathsf{ct}_{6}; 2^{j}\right)\right)$$

for *j*= log(*f*+1),…, log(*f*+1)+ log*n*−1. The output ciphertext is

$${}\mathsf{ct}_{7}=\texttt{Enc}\left[\begin{array}{ccc} \sum_{i} g\left(\mathbf{z}_{i}^{T}{\boldsymbol{\beta}}^{(t)}\right)\cdot z_{i0} & \cdots & \sum_{i} g\left(\mathbf{z}_{i}^{T}{\boldsymbol{\beta}}^{(t)}\right)\cdot z_{if} \\ \sum_{i} g\left(\mathbf{z}_{i}^{T}{\boldsymbol{\beta}}^{(t)}\right)\cdot z_{i0} & \cdots & \sum_{i} g\left(\mathbf{z}_{i}^{T}{\boldsymbol{\beta}}^{(t)}\right)\cdot z_{if} \\ \vdots & \ddots & \vdots \\ \sum_{i} g\left(\mathbf{z}_{i}^{T}{\boldsymbol{\beta}}^{(t)}\right)\cdot z_{i0} & \cdots & \sum_{i} g\left(\mathbf{z}_{i}^{T}{\boldsymbol{\beta}}^{(t)}\right)\cdot z_{if} \\ \end{array}\right],$$

as desired.

**Step 8:** For the learning rate *α*_*t*_, it uses the parameter *p*_*c*_ to compute the scaled learning rate $\phantom {\dot {i}\!}\Delta ^{(t)}= \lfloor {{2^{p_{c}}\cdot \alpha _{t}}}\rceil $. The public cloud updates ***β***^(*t*)^ using the ciphertext *ct*_7_ and the constant *Δ*^(*t*)^:

$$\begin{array}{*{20}l} &\mathsf{ct}_{8} \leftarrow \texttt{ReScale}\left(\Delta^{(t)}\cdot \mathsf{ct}_{7}; p_{c}\right),\\ &\mathsf{ct}_{\beta}^{(t+1)}\leftarrow \texttt{Add}\left(\mathsf{ct}_{\beta}^{(t)}, \mathsf{ct}_{8}\right). \end{array} $$

Finally it returns a ciphertext encrypting the updated modeling vector

$$\mathsf{ct}_{\beta}^{(t+1)}=\texttt{Enc}\left[\begin{array}{cccc} \beta_{0}^{(t+1)} & \beta_{1}^{(t+1)} & \cdots & \beta_{f}^{(t+1)} \\ \beta_{0}^{(t+1)} & \beta_{1}^{(t+1)} & \cdots & \beta_{f}^{(t+1)} \\ \vdots & \vdots & \ddots & \vdots \\ \beta_{0}^{(t+1)} & \beta_{1}^{(t+1)} & \cdots & \beta_{f}^{(t+1)} \end{array}\right].$$

where $\beta _{j}^{(t+1)}=\beta _{j}^{(t)}+\frac {\alpha _{t}}{n}\sum _{i} g\left (\mathbf {z}_{i}^{T}{\boldsymbol {\beta }}^{(t)}\right)\cdot z_{ij}$.

### Homomorphic evaluation of Nesterov’s accelerated gradient

The performance of leveled HE schemes highly depends on the depth of a circuit to be evaluated. The bottleneck of homomorphic evaluation of the GD algorithm is that we need to repeat the update of weight vector ***β***^(*t*)^ iteratively. Consequently, the total depth grows linearly on the number of iterations and it should be minimized for practical implementation.

For the homomorphic evaluation of Nesterov’s accelerated gradient, a clients sends one more ciphertext $\mathsf {ct}_{v}^{(0)}$ encrypting the initial vector **v**^(0)^ to the public cloud. Then the server uses an encryption *ct*_*z*_ of dataset *Z* to update two ciphertexts **v**^(*t*)^ and $\mathsf {ct}_{\beta }^{(t)}$ at each iteration. One can securely compute ***β***^(*t*+1)^ in the same way as the previous section. Nesterov’s accelerated gradient requires one more step to compute the second equation of (1) and obtain an encryption of **v**^(*t*+1)^ from $\mathsf {ct}_{\beta }^{(t)}$ and $\mathsf {ct}_{\beta }^{(t+1)}$.

**Step 9:** Let $\Delta ^{(t)}_{1} = \lfloor {{2^{p_{c}}\cdot \gamma _{t}}}\rceil $ and let $\Delta ^{(t)}_{2} = 2^{p_{c}}-\Delta ^{(t)}_{1}$. It obtains the ciphertext $\mathsf {ct}_{v}^{(t+1)}$ by computing

$$\begin{array}{*{20}l} \mathsf{ct}_{v}^{(t+1)} &\leftarrow \texttt{Add}\left(\Delta^{(t)}_{2}\cdot\mathsf{ct}_{\beta}^{(t+1)}, \Delta^{(t)}_{1}\cdot\mathsf{ct}_{\beta}^{(t)}\right),\\ \mathsf{ct}_{v}^{(t+1)} &\leftarrow \texttt{ReScale}\left(\mathsf{ct}_{v}^{(t+1)}; p_{c}\right). \end{array} $$

Then the output ciphertext is

$$\mathsf{ct}_{v}^{(t+1)}=\texttt{Enc}\left[\begin{array}{cccc} v_{0}^{(t+1)} & v_{1}^{(t+1)} & \cdots & v_{f}^{(t+1)} \\ v_{0}^{(t+1)} & v_{1}^{(t+1)} & \cdots & v_{f}^{(t+1)} \\ \vdots & \vdots & \ddots & \vdots \\ v_{0}^{(t+1)} & v_{1}^{(t+1)} & \cdots & v_{f}^{(t+1)} \end{array}\right],$$

which encrypts $v_{j}^{(t+1)}=(1 - \gamma _{t})\cdot \beta ^{(t+1)}_{j} + \gamma _{t}\cdot \beta ^{(t)}_{j}$ in the plaintext slots.

## Results

In this section, we present parameter sets with experimental results. Our implementation is based on the HEAAN library [21] that implements the approximate HE scheme of Cheon et al. [14]. The source code is publicly available at github [16].

### Parameters settings

We explain how to choose the parameter sets for the homomorphic evaluation of the (Nesterov’s) GD algorithm with security analysis. We start with the parameter *L* - the bitsize of a fresh ciphertext modulus. The modulus of a ciphertext is reduced after the ReScale operations and the evaluation of an approximate polynomial *g*(*x*).

The ReScale procedures after homomorphic multiplications (step 1 and 6) reduce the ciphertext modulus by *p* bits while the ReScale procedures after constant multiplications (step 3 and 8) require *p*_*c*_ bits of modulus reduction. Note that the ciphertext modulus remains the same for the step 9 for the Nesterov’s accelerated gradient if we compute step 8 and 9 together using some precomputed constants. We use a similar method with a previous work for the evaluation of the sigmoid function (see [13] for details); the ciphertext modulus is reduced by (2*p*+3) bits for the evaluation of *g*_3_(*x*), and (3*p*+3) bits for that of *g*_5_(*x*) and *g*_7_(*x*). Therefore, we obtain the following lower bound on the parameter *L*:

$$L=\left\{\begin{array}{ll} \text{\textsc{IterNum}} \cdot (3 p + 2 p_{c} + 3) + L_{0} & g = g_{3}, \\ \text{\textsc{IterNum}} \cdot (4 p + 2 p_{c} + 3) + L_{0} & g \in \{g_{5}, g_{7}\}, \end{array}\right. $$

where ITERNUM is the number of iterations of the GD algorithm and *L*_0_ denotes the bit size of the output ciphertext modulus. The modulus of the output ciphertext should be larger than 2^*p*^ in order to encrypt the resulting weight vector and maintain its precision. We take *p*=30, *p*_*c*_=20 and *L*_0_=35 in our implementation.

The dimension of a cyclotomic ring ${\mathcal {R}}$ is chosen as *N*=2^16^ following the security estimator of Albrecht et al. [22] for the learning with errors problem. In this case, the bit size *L* of a fresh ciphertext modulus should be bounded by 1284 to ensure the security level *λ*=80 against known attacks. Hence we repeat ITERNUM=9 iterations of GD algorithm *g*=*g*_3_, and ITERNUM=7 iterations when *g*=*g*_5_ or *g*=*g*_7_.

The smoothing parameter *γ*_*t*_ is chosen in accordance with [15]. The choice of proper GD learning rate parameter *α*_*t*_ normally depends on the problem at hand. Choosing too small *α*_*t*_ leads to a slow convergence, and choosing too large *α*_*t*_ could lead to a divergence, or a fluctuation near a local optima. It is often optimized by a trial and error method, which we are not available to perform. Under these conditions harmonic progression seems to be a good candidate and we choose a learning rate $\alpha _{t} = \frac {10}{t+1}$ in our implementation.

### Implementation

All the experimentations were performed on a machine with an Intel Xeon CPU E5-2620 v4 at 2.10 GHz processor.

**Task for the iDASH challenge.** In genomic data privacy and security protection competition 2017, the goal of Track 3 was to devise a weight vector to predict the disease using the genotype and phenotype data (Additional file 1: iDASH). This dataset consists of 1579 samples, each of which has 18 features and a cohort information (disease vs. healthy). Since we use the ring dimension *N*=2^16^, we can only pack up to *N*/2=2^15^ dataset values in a single ciphertext but we have totally 1579×19>2^15^ values to be packed. We can overcome this issue by dividing the dataset into two parts of sizes 1579×16 and 1579×3 and encoding them separately into two ciphertexts. In general, this method can be applied to the datasets with any number of features: the dataset can be encrypted into ⌈(*f*+1)·*n*·(*N*/2)^−1^⌉ ciphertexts.

In order to estimate the validity of our method, we utilized 10-fold cross-validation (CV) technique: it randomly partitions the dataset into ten folds with approximately equal sizes, and uses every subset of 9 folds for training and the rest one for testing the model. The performance of our solution including the average running time per fold of 10-fold CV (encryption and evaluation) and the storage (encrypted dataset) are shown in Table 1. This table also provides the average accuracy and the AUC (Area Under the Receiver Operating Characteristic Curve) which estimate the quality of a binary classifier.

**Table 1:** Implementation results for iDASH dataset with 10-fold CV

Sample	Feature	deg*g*	Iter	Enc	Learn	Storage	Accuracy	AUC
num	num		num	time	time			
1579	18	3	9	4s	7.94 min	0.04 GB	61.72%	0.677
		5	7	4s	6.07 min	0.04 GB	62.87%	0.689
		7	7	4s	7.01 min	0.04 GB	62.36%	0.689

**Comparison** We present some experimental results to compare the performance of implementation to [13]. For a fair comparison, we use the same 5-fold CV technique on five datasets - the Myocardial Infarction dataset from Edinburgh [23] (Additional file 2: Edinburgh), Low Birth Weight Study (Additional file 3: lbw), Nhanes III (Additional file 4: nhanes3), Prostate Cancer Study (Additional file 5: pcs), and Umaru Impact Study datasets (Additional file 6: uis) [24–27]. All datasets have a single binary outcome variable.

All the experimental results are summarized in Table 2. Our new packing method could reduce the storage of ciphertexts and the use of Nesterov’s accelerated gradient achieves much higher speed than the approach of [13]. For example, it took 3.6 min to train a logistic regression model using the encrypted Edinburgh dataset of size 0.02 GB, compared to 114 min and 0.69 GB of the previous work [13], while achieving the good qualities of the output models.

**Table 2:** Implementation results for other datasets with 5-fold CV

Dataset	Sample	Feature	Method	deg*g*	Iter	Enc	Learn	Storage	Accuracy	AUC
	num	num			num	time	time			
Edinburgh	1253	9	Ours	5	7	2s	3.6 min	0.02 GB	91.04%	0.958
			[13]	3	25	12s	114 min	0.69 GB	86.03%	0.956
			[13]	7	20	12s	114 min	0.71 GB	86.19%	0.954
lbw	189	9	Ours	5	7	2s	3.3 min	0.02 GB	69.19%	0.689
			[13]	3	25	11s	99 min	0.67 GB	69.30%	0.665
			[13]	7	20	11s	86 min	0.70 GB	69.29%	0.678
nhanes3	15649	15	Ours	5	7	14s	7.3 min	0.16 GB	79.22%	0.717
			[13]	3	25	21s	235 min	1.15 GB	79.23%	0.732
			[13]	7	20	21s	208 min	1.17 GB	79.23%	0.737
pcs	379	9	Ours	5	7	2s	3.5 min	0.02 GB	68.27%	0.740
			[13]	3	25	11s	103 min	0.68 GB	68.85%	0.742
			[13]	7	20	11s	97 min	0.70 GB	69.12%	0.750
uis	575	8	Ours	5	7	2s	3.5 min	0.02 GB	74.44%	0.603
			[13]	3	25	10s	104 min	0.61 GB	74.43%	0.585
			[13]	7	20	10s	96 min	0.63 GB	75.43%	0.617

## Discussion

The rapid growth of computing power initiated the study of more complicated ML algorithms in various fields including biomedical data analysis [28, 29]. HE system is a promising solution for the privacy issue, but its efficiency in real applications remains as an open question. It would be great if we could extend this work to other ML algorithms such as deep learning.

One constraint in our approach is that the number of iterations of GD algorithm is limited depending on the choice of HE parameter. In terms of asymptotic complexity, applying the bootstrapping method of approximate HE scheme [30] to the GD algorithm would achieve a linear computation cost on the iteration number.

## Conclusion

In the paper, we presented a solution to homomorphically evaluate the learning phase of logistic regression model using the gradient descent algorithm and the approximate HE scheme. Our solution demonstrates a good performance and the quality of learning is comparable to the one of an unencrypted case. Our encoding method can be easily extended to a large-scale dataset, which shows the practical potential of our approach.

## Additional files

Additional file 1 iDASH. iDASH challenge dataset (TXT 59 kb)

Additional file 2 Edinburgh. The Myocardial Infarction dataset from Edinburgh (TXT 24 kb)

Additional file 3 lbw. Low Birth Weight Study dataset (TXT 4 kb)

Additional file 4 nhanes3. Nhanes III dataset (TXT 745 kb)

Additional file 5 pcs. Prostate Cancer Study dataset (TXT 9 kb)

Additional file 6 uis. Umaru Impact Study dataset (TXT 11 kb)

## Abbreviations

AUC: Area under the receiver operating characteristic curve
CV: Cross validation
GD: Gradient descent
HE: Homomorphic encryption
ML: Machine learning
